# Dual Function Modification of Cs_2_CO_3_ for Efficient Perovskite Solar Cells

**DOI:** 10.3390/nano12183144

**Published:** 2022-09-10

**Authors:** Debei Liu, Qingxin Zeng, Cunyun Xu, Hongfei Liang, Lijia Chen, Qunliang Song

**Affiliations:** 1 Institute for Clean Energy & Advanced Materials, Faculty of Materials and Energy, Southwest University, Chongqing 400715, China; 2 Chongqing Yufu Holding Group Co., Ltd., Chongqing 400715, China; 3 College of International Studies, Southwest University, Chongqing 400715, China; 4 College of Physics and Electronics Engineering, Chongqing Normal University, Chongqing 401331, China

**Keywords:** solar cells, optical materials, thin films, electrical properties, interface structure

## Abstract

Organic-inorganic hybrid perovskite solar cells (PeSCs) attract much attention in the field of solar cells due to their excellent photovoltaic performance. Many efforts have been devoted to improving their power conversion efficiency (PCE). However, few works focus on simultaneously improving their electrical and optical property. Herein, a simple strategy is proposed to improve the PCE from 19.8% of a reference device to 22.9%, by utilizing cesium carbonate (Cs_2_CO_3_) to modify indium tin oxide (ITO) substrate. The insertion of a Cs_2_CO_3_-modification layer between ITO substrate and SnO_2_ electron transport layer simultaneously offers two benefits: improving the electron extraction capability and adjusting the light field distribution in the device. The optical optimization effect of Cs_2_CO_3_ revealed in this work has not been reported before. This work provides a new and simple strategy to obtain high performance PeSCs by improving the electrical and optical properties of the devices at the same time.

Organic-inorganic hybrid halide perovskite solar cells (PeSCs) have greatly impacted the field of solar cells and have received a lot of attention from researchers. The high PCE of up to 25.6% is derived from the suitable and adjustable optical band gap, high light absorption coefficient, low exciton binding energy, direct band gap, long carrier diffusion length, high defect tolerance of perovskite materials and so on. Researchers have done much work and proposed various strategies, especially for interface modification to improve the performance of PeSCs [1,2,3]. These efforts promoted the device performance with an internal quantum efficiency (IQE) approaching 100%, which means that the electrical performance of the device is almost impossible to improve [4,5,6]. However, there is still a gap between the current device PCE and the theoretical limit (31% according to the detailed balance model) [7]. An analysis by a revised detailed balance model pointed out that optical loss can account for 40% of total energy loss [8]. Therefore, reducing the optical loss while improving the electrical properties of the device at the same time could provide an alternative strategy to further enhance the PCE of PeSCs, where little attention has been paid.

Cesium carbonate (Cs_2_CO_3_) is an excellent interface modification material widely used in organic light emitting diodes (OLED), organic solar cells (OSCs), organic field-effect transistors (OFET) and other devices. Cs_2_CO_3_ can also be used as an interfacial passivation layer for ETL/perovskite to improve the efficiency of perovskite solar cells by reducing the recombination rate at the interface [9]. We have used Cs_2_CO_3_ to electrically modify the electron transport layer (ETL) of p-i-n type PeSCs and improved device efficiency [10]. On the other hand, due to the different refractive index of Cs_2_CO_3_ from indium tin oxide (ITO) and tin oxide (SnO_2_), it is possible to use Cs_2_CO_3_ to manage optical properties of SnO_2_-based PeSCs while utilizing its electrical advantages. In this work, Cs_2_CO_3_ is inserted between the ITO substrate and SnO_2_ ETL to modify both electrical and optical properties of n-i-p type PeSCs. Thanks to the Cs_2_CO_3_ modification, all the short-circuit current density (J_sc_), open-circuit voltage (V_oc_) and fill factor (FF) are improved, leading to a high PCE of 22.9%. The greatly improved J_sc_ and then external quantum efficiency (EQE) are ascribed to the Cs_2_CO_3_ modification induced by electrical and optical improvements. Moreover, the better electrical properties (the higher carrier extraction capability) with Cs_2_CO_3_ modification in the device is considered to be the reason for the improvement of V_oc_ and FF. The better optical properties induced by Cs_2_CO_3_ modification is the main reason for the enhanced J_sc_.

Here, the n-i-p type PeSCs with a structure of ITO/SnO_2_/perovskite (FA_0.9_MA_0.1_PbI_3_)/Spiro-OMeTAD/Ag is selected as a reference device (see Supplementary Material Note 1 for details) [11]. As mentioned above, a Cs_2_CO_3_ buffer layer is inserted between ITO and SnO_2_ to get a final structure of ITO/Cs_2_CO_3_/SnO_2_/perovskite/Spiro-OMeTAD/Ag. To obtain the best performance in the Cs_2_CO_3_-modification device, an optimization process has been conducted by changing the concentration of Cs_2_CO_3_ solution, as shown in Figure S1. Considering the performance and solubility, a 40 mg/mL solution of Cs_2_CO_3_ in ethanol is finally selected in this work. The champion Cs_2_CO_3_-modification device is compared with the reference device in Figure 1 (with corresponding EQE results shown in Figure S2). The Cs_2_CO_3_-modification device exhibits a *PCE* of 22.9%, a *V_OC_* of 1.19 V, a *J_SC_* of 24.49 mA·cm^−2^ and a *FF* of 0.78. In comparison, a *PCE* of 19.8% is observed in the reference device with a *V_OC_* of 1.15 V, a *J_SC_* of 22.83 mA·cm^−2^ and a *FF* of 0.75. Obviously, the PCE is significantly improved due to all parameter (*V_OC_*, *J_SC_* and *FF*) enhancements in the Cs_2_CO_3_-modification device compared to the reference device with the greatest contribution from the *J_SC_* (from 22.83 mA·cm^−2^ of the reference device to 24.49 mA·cm^−2^, with ~7.2% enhancement). The stabilized photocurrent measurement at the maximum power point (MPP) and hysteresis test are performed and shown in Figures S3 and S4, respectively.

The increased V_oc_ and FF should be attributed to the improvement of carrier extraction of the device brought by Cs_2_CO_3_ modification, which is verified by transient photocurrent decay (TPC) tests. The TPC test is conducted under short circuit condition by recording the decay of photocurrent caused by a pulsed laser. Thus, the TPC signal reflects the carrier extraction capacity inside the device. In general, the faster the TPC signal decays, the easier it is for carriers in the device to be collected for a given laser intensity [12]. The normalized TPCs of the two devices are compared in Figure 2. Clearly, the decay time, defined as the time scale from the maximum current to no current, of the reference and Cs_2_CO_3_-modification device are ~40 and 10 ns, respectively. Thus, the Cs_2_CO_3_-modification device shows a faster decay behavior compared to the reference device, which suggests a much higher extraction capability of charge carriers in the Cs_2_CO_3_-modified device. It should be highlighted that the protrusion in the TPC curve at ~6 ns is attributed to the broadening of the laser itself. The corresponding J-V characteristics displayed in Figure S5 reflects the improvement of electrical performance of ITO/SnO_2_ substrate after Cs_2_CO_3_ modification, which should be the reason for the improved carrier extraction ability. Two samples with the structure of ITO/(with or without) Cs_2_CO_3_/SnO_2_/PEDOT:PSS/MoO_3_/Ag have been designed to verify the electrical advantage of Cs_2_CO_3_ modification. As evidenced in Figure S5, the Cs_2_CO_3_-modification layer increases the current at the positive bias and decreases the current at the negative bias, which suggests a relative high electron extraction capability and a low leakage current in the PeSCs device after Cs_2_CO_3_ modification. The improved carrier extraction ability may be attributed to the effect of Cs_2_CO_3_ on the work function of ITO and the smoother and denser SnO_2_ layer after Cs_2_CO_3_ modification. A large number of references have proven that Cs_2_CO_3_ can reduce the work function of the ITO substrate, so that electrons are more easily transferred from SnO_2_ to ITO [13]. In addition to that, the introduction of Cs_2_CO_3_ improves the wettability of the ITO substrate to the SnO_2_ solution, thus making the prepared SnO_2_ film more dense and flat, as shown in Figures S6 and S7. However, these small changes are not reflected in the morphology and then the properties of perovskite layer on SnO_2_. SEM images of perovskite films in Figure S8 and transient photovoltage decay (TPV) test results in Figure S9 prove that positive effects of Cs_2_CO_3_ to the device performance are not realized via changing the perovskite layer. In addition, XPS tests on the surface of the sample ITO/Cs_2_CO_3_/SnO_2_ show that SnO_2_ effectively prevents the diffusion of Cs to the perovskite, which prove that the effect of Cs_2_CO_3_ modification is not caused by the incorporation of Cs elements into perovskite, as shown in Figure S10.

The improvement of J_sc_ after Cs_2_CO_3_ modification is not only related to the improvement of the electrical performance of the device, but also to the increase of the transmittance of the substrate. The transmittance changes of ITO and ITO/SnO_2_ substrate after CS_2_CO_3_ modification is obtained using a Shimadzu UV-2550 Spectrophotometer (Shimadzu, Kyoto, Japan), with the optical path shown in Figure S11. As shown in Figure 3, the transmittance ratios of both ITO and ITO/SnO_2_ substrates before and after CS_2_CO_3_ modification are greater than 1 at almost all wavelengths, which means that CS_2_CO_3_ greatly improves the transmission of the substrate. The matrix optical calculation results of the device confirm this observation. The structure of Air/glass (700,000 nm)/SiO_2_ (30 nm)/ITO (135 nm)/SnO_2_ or Cs_2_CO_3_/SnO_2_ (SnO_2_ for 45 nm and Cs_2_CO_3_/SnO_2_ for 50 nm)/perovskite (600 nm)/Spiro-OMeTAD (135 nm)/Ag (100 nm) is used in the optical simulation, as shown in Figure S12 (the details of calculation program and parameters are given in the Supplementary Material Note 2). The thickness of each layer was verified by cross-sectional SEM as shown in Figure S13. The optical constants of SnO_2_ or Cs_2_CO_3_/SnO_2_ layers were obtained by an ellipsometer, and the optical constants of other layers were given in the literature [14,15,16]. All optical constants are shown in the spreadsheet file “Optical Constants” in the Supplementary Material. After calculation, the light absorptions of the perovskite layer before and after Cs_2_CO_3_ modification are shown in Figure 4a. Figure 4b shows the ratio of light absorption of the perovskite layer after Cs_2_CO_3_ modification divided by the one before Cs_2_CO_3_ modification. It can be seen that the light absorption of the perovskite layer in the 300–800 nm wavelength range after Cs_2_CO_3_ modification has been enhanced to certain degrees. SEM results show that this increase in transmittance may be due to the unique structure of the Cs_2_CO_3_ layer after being washed by SnO_2_ solution. As shown in Figure S14, a continuous layer with some pinholes is formed on ITO after spin-coating Cs_2_CO_3_. Then, the same amount of deionized water as the SnO_2_ solution used in preparing SnO_2_ layer was spin-coated on Cs_2_CO_3_ to mimic the real experimental condition. It is found that the Cs_2_CO_3_ layer changes to a discontinuous distribution, which is also confirmed by energy dispersive X-ray spectroscopy (EDS) test, which can be the reason for the increased optical transmission of the substrate, as shown in Figure S15.

In summary, we have fabricated a high efficiency PeSC device by modifying the ITO substrate with the Cs_2_CO_3_ layer. The *PCE* increases from 19.8% of the reference device to 22.9% of the Cs_2_CO_3_-modification device. Through TPV, TPC, AFM, UV-vis, etc. measurement, the roles of the Cs_2_CO_3_-modification layer have been addressed: improving the electron extraction capability and adjusting the light-field distribution in the perovskite layer at the same time. The improved electron extraction capability is presumably from the more compact and denser SnO_2_ layer after Cs_2_CO_3_-modification and work function change of ITO substrate, thus contributing a part improvement of *J_sc_*, *V_oc_*, and *FF* in the device. On the other hand, the light field distribution adjustment is beneficial for utilizing more photons in the perovskite layer due to enhanced transmission of the ITO substrate. Undoubtedly, this work provides a new strategy to improve the *PCE* of PeSCs devices by simultaneously enhancing the electrical and optical properties, which has rarely been reported before.

## Figures and Tables

**Figure 1:**
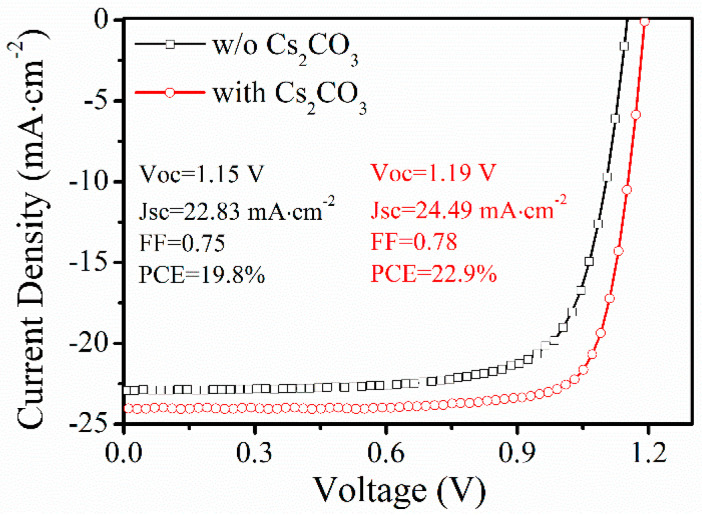
The current-voltage characteristics of the device with structure ITO/(with and without) Cs_2_CO_3_/SnO_2_/FA_0.9_MA_0.1_PbI_3_/Spiro-OMeTAD/Ag.

**Figure 2:**
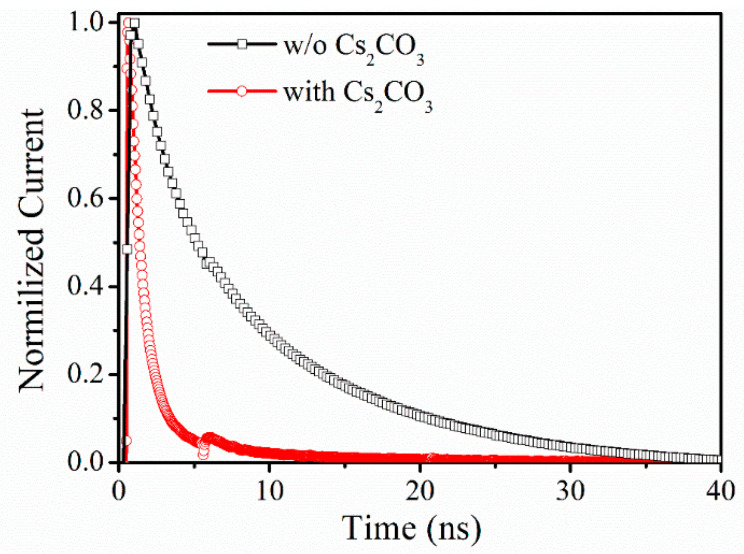
The TPC tests of the reference and the Cs_2_CO_3_-modification devices.

**Figure 3:**
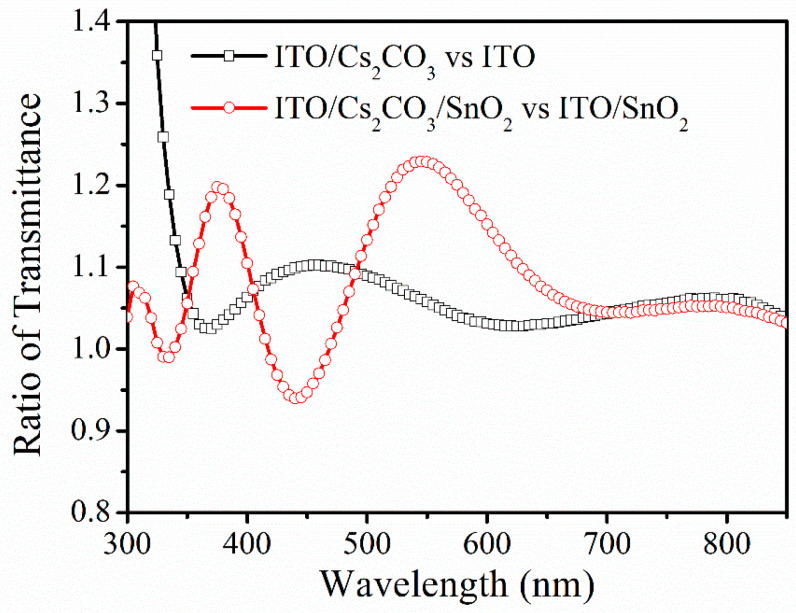
The transmittance changes of ITO and ITO/SnO_2_ substrate after CS_2_CO_3_ modification.

**Figure 4:**
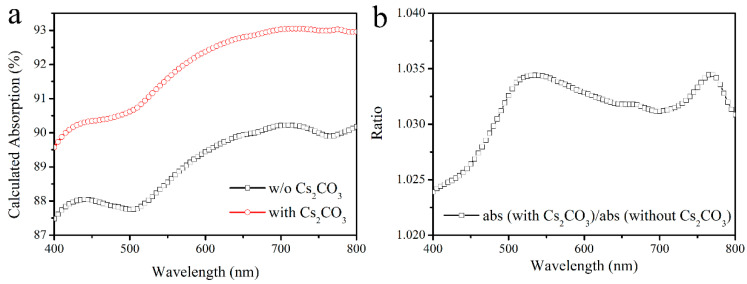
(**a**) The calculated absorption of perovskite layer in devices with or without Cs_2_CO_3_ modification. (**b**) The result of calculated light absorption of perovskite layer with Cs_2_CO_3_ modification divided by light absorption of the unmodified one.

## Supplementary Materials

The following supporting information can be downloaded at: https://www.mdpi.com/article/10.3390/nano12183144/s1, Figure S1: The data statistics of VOC, JSC, FF and PCE; Figure S2: The EQE and Jin results; Figure S3: The stabilized photocurrent measurement at the maximum power point (MPP) of the device; Figure S4: The hysteresis tests of the devices; Figure S5: J-V curves test; Figure S6: Contact angle test results; Figure S7: AFM results; Figure S8: SEM images of the perovskite layer; Figure S9: The TPV tests results; Figure S10: XPS tests results; Figure S11: The optical path diagram; Figure S12: The device optical structure; Figure S13: Cross-sectional SEM images of the device; Figure S14: The SEM images; Figure S15: The EDS image; Supplementary Material Note 1: Device fabrication and test methods; Note 2: Optical transmission matrix calculation details.
