# Supplementary material for: Dual Function Modification of Cs2CO3 for Efficient Perovskite Solar Cells
Source: Nanomaterials (Basel). 2022 Sep 10;12(18):3144. doi: 10.3390/nano12183144 (PMC9504462; doi:10.3390/nano12183144)
Supplement: Supplementary file 1 [file nanomaterials-12-03144-s001.zip › nanomaterials-1896991-supplementary.pdf]

nanomaterials

Supplementary Materials

Dual Function Modification of Cs_2CO_3 for Efficient Perovskite Solar Cells

Debei Liu [1,2,*], Qingxin Zeng [1], Cunyun Xu [1], Hongfei Liang [3], Lijia Chen [4] and Qunliang Song [1,*]

[1] Institute for Clean Energy & Advanced Materials, Faculty of Materials and Energy, Southwest University, Chongqing 400715, China
[2] Chongqing Yufu Holding Group Co., Ltd., Chongqing 400715, China
[3] College of International Studies, Southwest University, Chongqing 400715, China
[4] College of Physics and Electronics Engineering, Chongqing Normal University, Chongqing 401331, China
* Correspondence: dbliu1992@foxmail.com (D.L.); qlsong@swu.edu.cn (Q.S.)

Note 1

Method

Materials. All materials used in the experiments are commercially available materi-als. Materials used to prepare perovskite films, including lead iodide (PbI_2), methylammo-nium iodide (MAI), formamidinium Iodide (FAI) were bought from Xi'an Polymer Light Technology Corporation (Xi’an, China). The material used to prepare the device, [6,6]−phenyl−C61−butyric acid methyl ester (PCBM), Poly(3,4 −ethylenedioxythiophene) polystyrene sulfonate (PEDOT:PSS) and 2,2',7,7'−Tetrakis[N,N−di(4−methoxy-phenyl)amino]−9,9'−spirobifluorene (Spiro −OMeTAD) were also purchased from Xi'an Polymer Light Technology Corporation. Tin (IV) oxide, 15% in H_2O colloidal dispersion used to prepare SnO_2 film was obtained from Thermo Fisher Scientific (Shanghai, China). Cesium carbonate (Cs_2CO_3) was purchased from Sigma−Aldrich (Darmstadt, Germany). The solvents used in the experiments, including N,N−Dimethylformamide (DMF), dime-thyl sulfoxide (DMSO), isopropanol (IPA), and chlorobenzene (CB), were all obtained from J&K Scientific Ltd (Newark, Germany).

Device fabrication. The structure of the perovskite solar cells used in this work is ITO/ (with or without) Cs_2CO_3/SnO_2/$FA_{0.9}MA_{0.1}PbI_3$/Spiro−OMeTAD/Ag, and the device was fabricated according to the following procedure. The first step is the cleaning of the ITO substrate, which is sequentially cleaned by a mild detergent of Decon 90, the distilled wa-ter and the ethanol in ultrasonic machine (KQ3200DV, Shumei, Kunshan, China), fol-lowed by drying with nitrogen flow. In the second step, for Cs_2CO_3 modified devices, the Cs_2CO_3 film is spin−coated on ITO at a speed of 3000 rpm. The third step is to spin−coat the film with SnO_2 solution diluted to 7.5% weight ratio at 4000 rpm, and anneal the spin−coated SnO_2 film at 150 °C for 30 min. The fourth step is to spin−coat the lead iodide (PbI_2) layer. 645.414 mg of PbI_2 is dissolved in a mixture of 800 μL of DMF and 200 μL of DMSO, and then spin coated onto the SnO_2 layer at 4000 rpm. The fifth step is to spin−coat $FA_{0.9}MA_{0.1}I$ solution on the PbI_2 film at 1700 rpm, where $FA_{0.9}MA_{0.1}I$ solution is obtained by dissolving 45 mg FAI and 5 mg MAI in 1 mL IPA. The sixth step is to wash the wet film mixture of $FA_{0.9}MA_{0.1}I$ solution and PbI_2 with IPA, and take 200 μL of IPA quickly to drip when the mixed film is spun to 4000 rpm. For devices that require repeated $FA_{0.9}MA_{0.1}I$ solution, repeat steps 4 and 5. After that, the mixed film is annealed at 60 °C for 30 min, and the preparation of the perovskite film was completed. The seventh step is to spin−coat the Spiro−OMeTAD film. The used solution is dissolved 72.5 mg Spiro−OMeTAD in 1 mL CB with appropriate additives as reported in the previous literature [17]. The spin−coated Spiro−OMeTAD film needs to be oxidized in an electronic drying oven for more than 12 h. Finally, a 100 nm thick Ag electrode is evaporated to complete the device fabrication.

The preparation of samples without perovskite layer with the structure of ITO/ (with or without) Cs_2CO_3/SnO_2/PEDOT:PSS/MoO_3/Ag is fabricated according to the following process. First, clean the ITO substrate. Second, spin−coat the Cs_2CO_3 solution on ITO at a speed of 3000 rpm. Third, spin−coat the film with SnO_2 solution and anneal it at 150 °C for 30 min. Fourth, spin−coat PEDOT:PSS at a speed of 6000 rpm. Fifth, deposit 10 nm thick MoO_3 layer. Finally, a 100 nm thick Ag electrode is deposited.

Characterization. The SEM image in this paper was obtained by scanning electron microscopy (JSM−6700F, Tokyo, Japan). The current−voltage characteristics curves were taken from Keithley 2400 source meter under illumination of 100 mW·cm^{-2} simulated light from a Newport simulator (model 94043A, Bozeman, Malta) with a scan window from 1.2 to −1.2 V (reverse) or −1.2 to 1.2 V (forward) with a voltage step of 10 mV and 0 ms delay time. The stabilized photocurrent measurement at the maximum power point was tested with Keithley by continuously recording current at a certain bias voltage. The EQE test was performed by an assembled system consisting of a Xenon arc lamp (Newport, 350 W, 66902, Bozeman, Malta) as a light source, a monochromatic instrument (Newport, 74125, Bozeman, Malta) as spectrometer, and a lock−in amplifier (Newport, 70104, Bozeman, Malta) for signal collection. The UV−Vis transmission spectra were acquired using Shimadzu UV−2550 Spectrophotometer (Kyoto, Japan). The transient photocurrent/photovoltage decay (TPC/TPV) measurements were conducted with a home−built equipment as described in reference [18].

Note 2

The optical field distribution in the device was simulated using OPTICAL software (Version 2, Boston, MA, USA), and the device structure is Air/glass (700,000 nm)/SiO_2 (30 nm)/ITO (135 nm)/SnO_2 (Cs_2CO_3/SnO_2)(SnO_2 for 45 nm and Cs_2CO_3/SnO_2 for 50 nm) /perovskite (600 nm)/Spiro−OMeTAD (135 nm)/Ag (100 nm). OPTICAL is a GPL optical simulation program written by Python, wxWindows, wxPython and Boa Constructor, which can be obtained from numpy.scipy.org. Thickness of each layer was obtained by Step Profiler or SEM cross section.

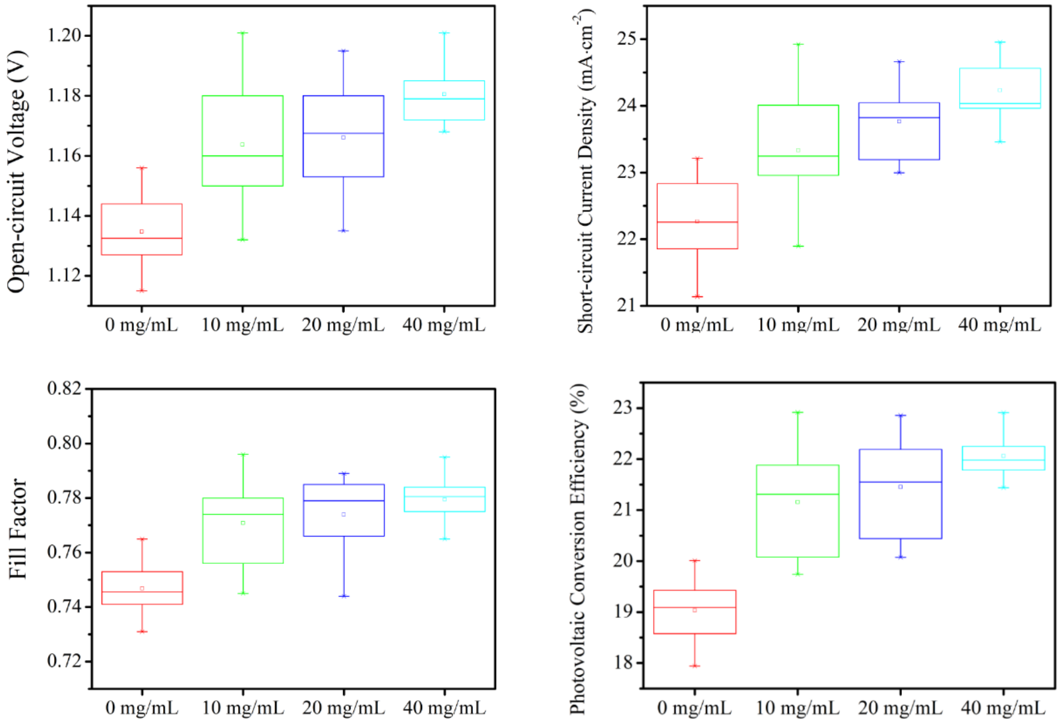

Figure S1. The data statistics of open−circuit voltage (V_{oc}), short−circuit current density (J_{sc}), fill factor (FF) and photovoltaic conversion efficiency (PCE) of device with different Cs_2CO_3 concentrations, where the abscissas is the concentration of Cs_2CO_3.

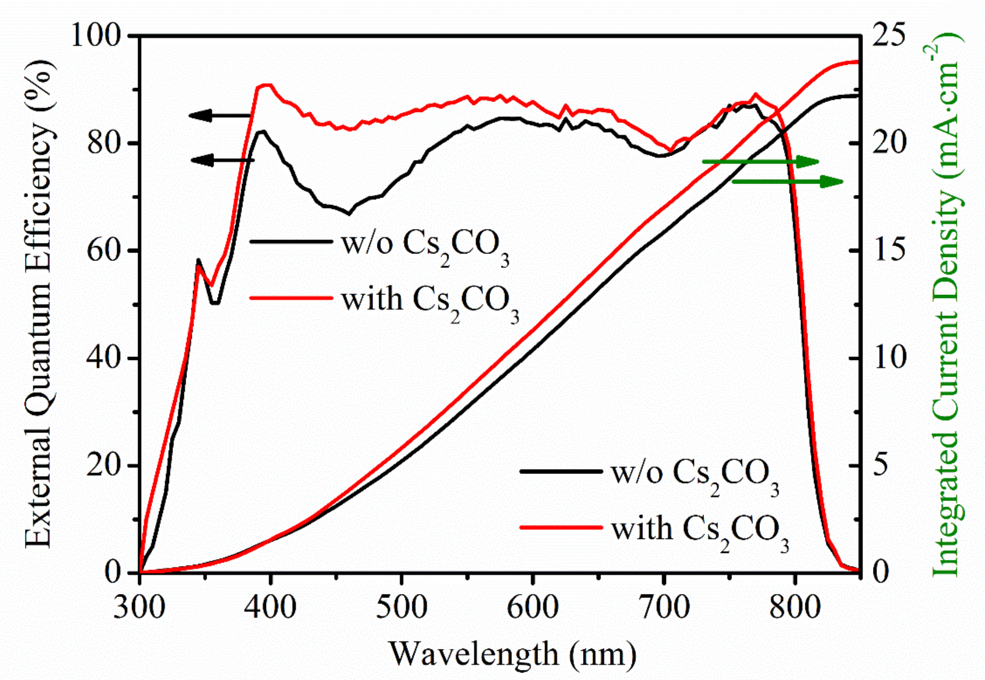

Figure S2. The EQE and J_{in} results of the devices with and without Cs_2CO_3 modification.

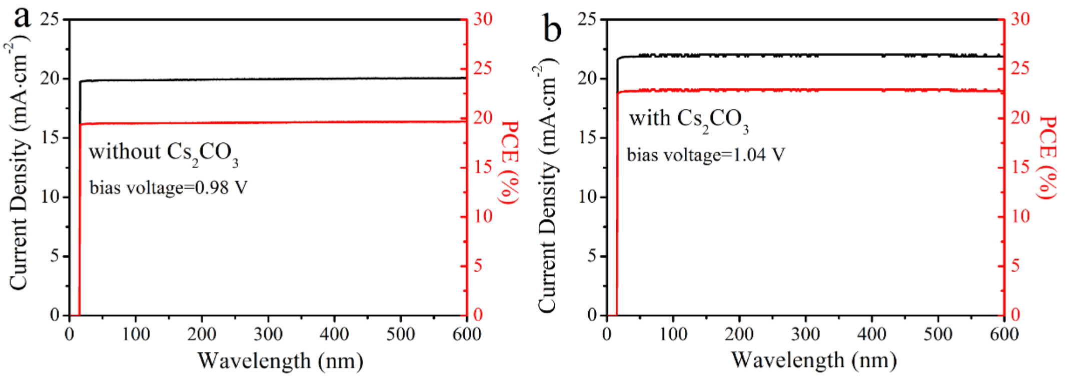

Figure S3. The stabilized photocurrent measurement at the maximum power point (MPP) of the device (**a**) without and (**b**) with Cs_2CO_3 modification.

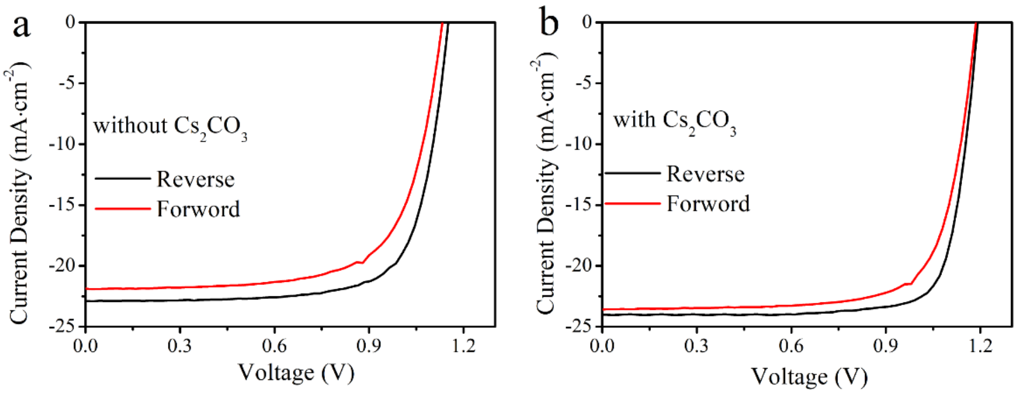

Figure S4. The hysteresis tests of the devices (**a**) without and (**b**) with Cs_2CO_3 modification.

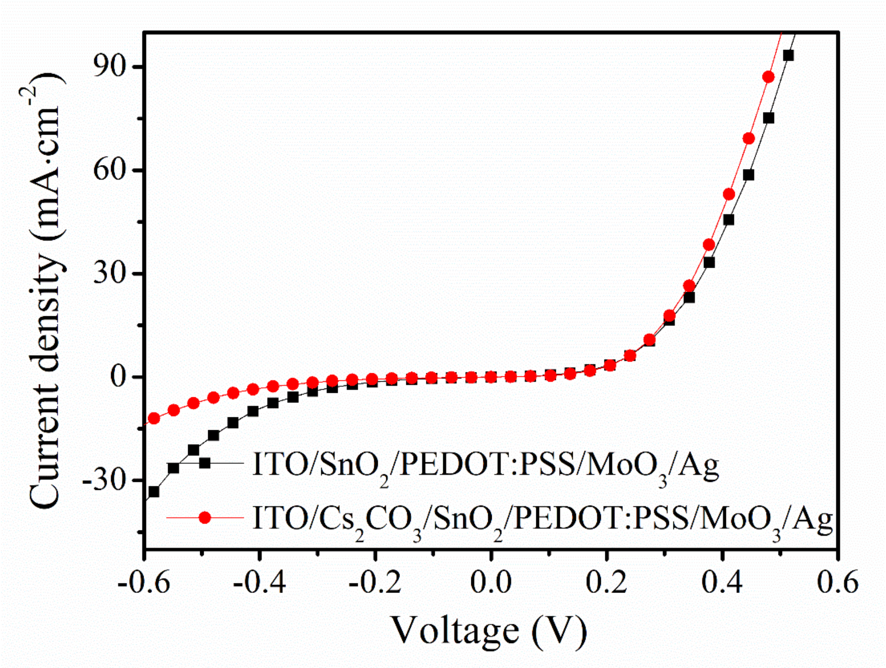

Figure S5. J–V curves of samples with the structure of ITO/ (with and without) Cs_2CO_3/SnO_2/PEDOT:PSS/MoO_3/Ag. The ITO is connected to the cathode of the source meter.

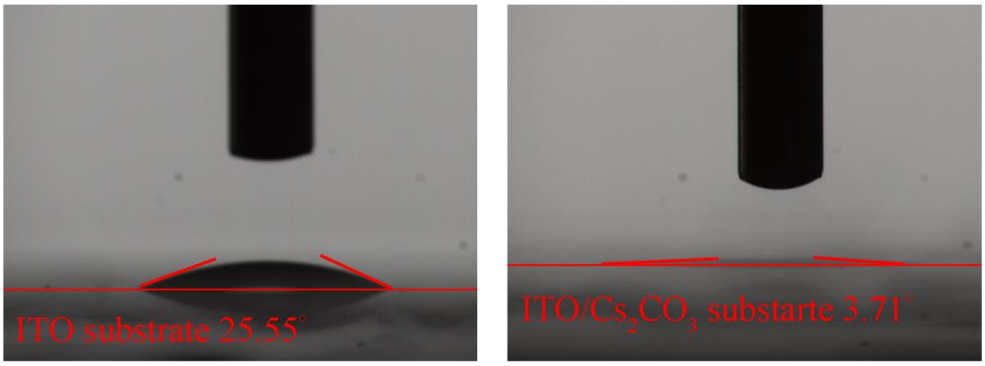

Figure S6. Contact angle test results of ITO and ITO/Cs_2CO_3 substrate. The ITO substrate modified with Cs_2CO_3 shows better wettability to SnO_2 solution.

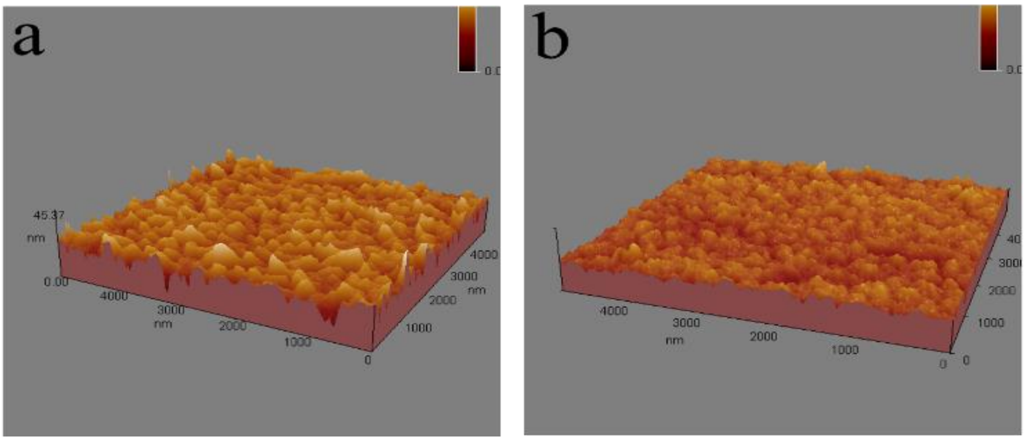

Figure S7. AFM results of SnO_2 film on (**a**) bare ITO and (**b**) Cs_2CO_3–modification ITO substrates. The SnO_2 film formed on ITO/Cs_2CO_3 substrate shows a denser and smaller roughness (root mean square roughness, σ_{rms} ~ 1.3 nm) when compared to that of bare ITO case (σ_{rms} ~ 3.7 nm). It should be noted that the σ_{rms} here is extracted from the software provided by the instrument itself and the σ_{rms} given here is indicative only.

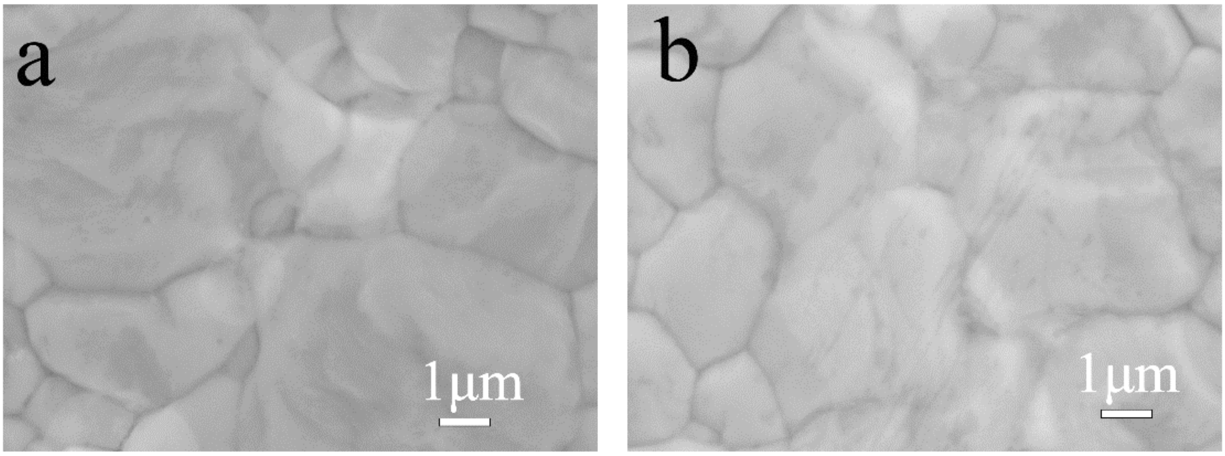

Figure S8. SEM images of the perovskite layer in the samples of (**a**) ITO/SnO_2/perovskite and (**b**) ITO/Cs_2CO_3/SnO_2/perovskite. The images show that the morphologies of the two samples are similar.

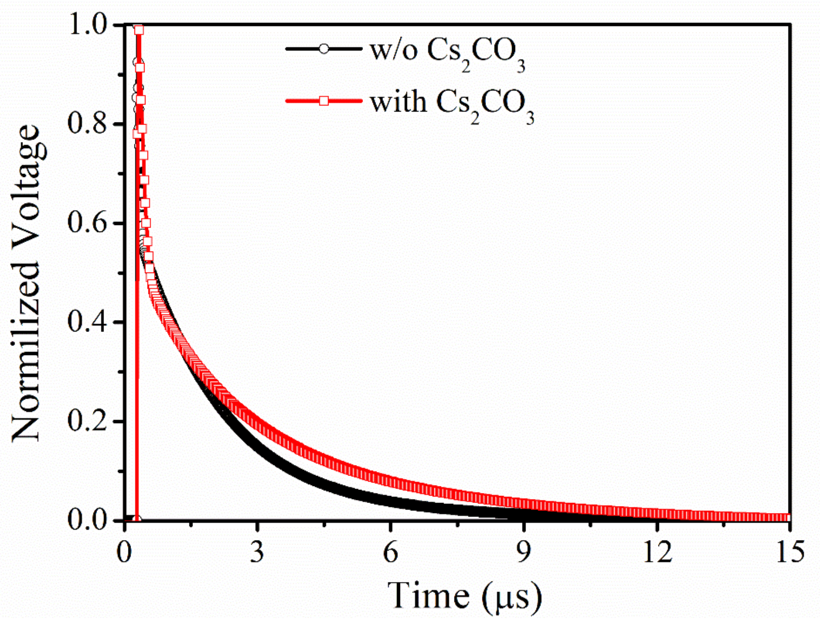

Figure S9. The TPV tests of the device with and without Cs_2CO_3 modification.

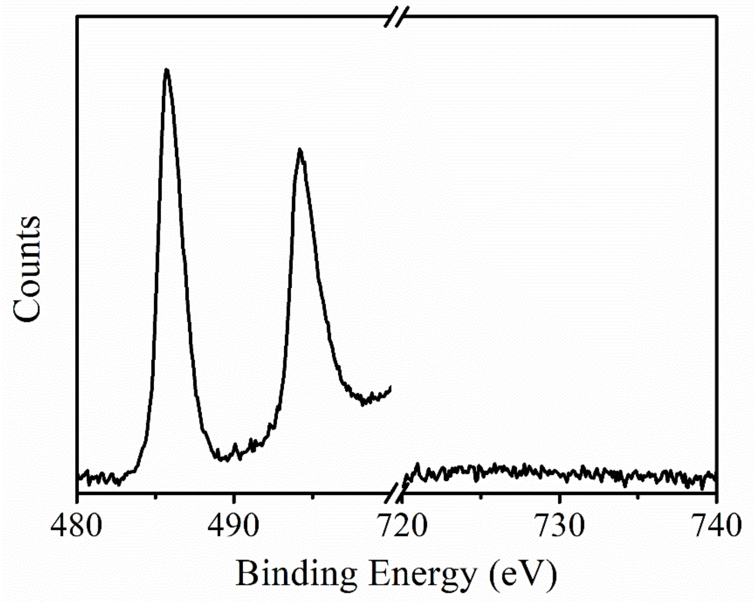

Figure S10. XPS tests on the surface of the device ITO/Cs_2CO_3/SnO_2. The 485 and 494 eV peaks can be attributed to the Sn element in SnO_2, while the 724 eV position of the Cs element does not show the peak position, which means that there is no Cs element on the surface of the ITO/Cs_2CO_3/SnO_2 device.

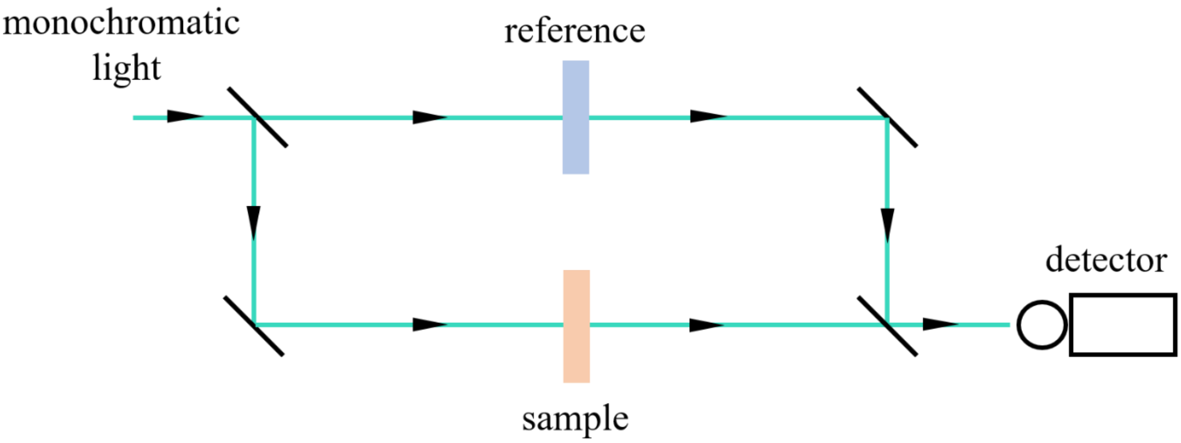

Figure S11. The optical path diagram when the transmittance change test is performed, where the reference is ITO or ITO/SnO_2, and the samples are ITO/Cs_2CO_3 and ITO/Cs_2CO_3/SnO_2, respectively.

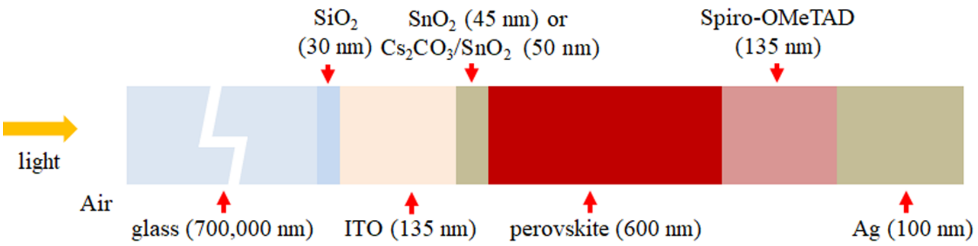

Figure S12. The device structure with or without Cs_2CO_3 modification used in optical simulation.

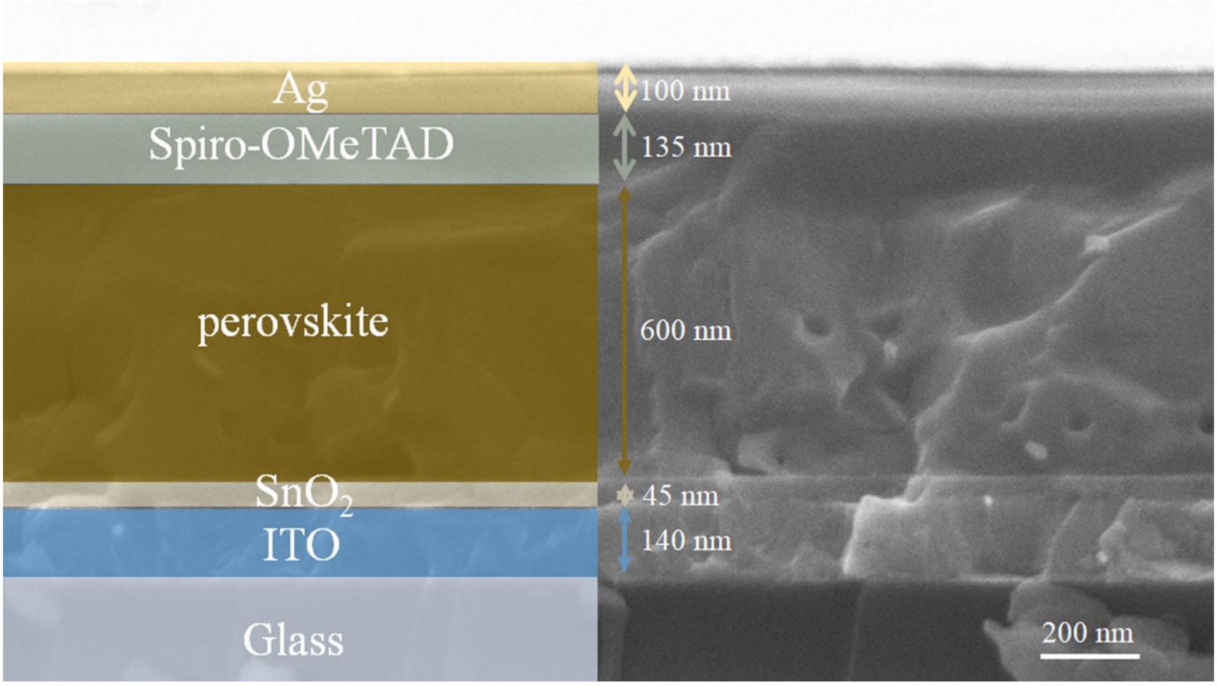

Figure S13. Cross–sectional SEM images of the device with structure ITO/SnO_2/perovskite/Spiro–OMeTAD/Ag.

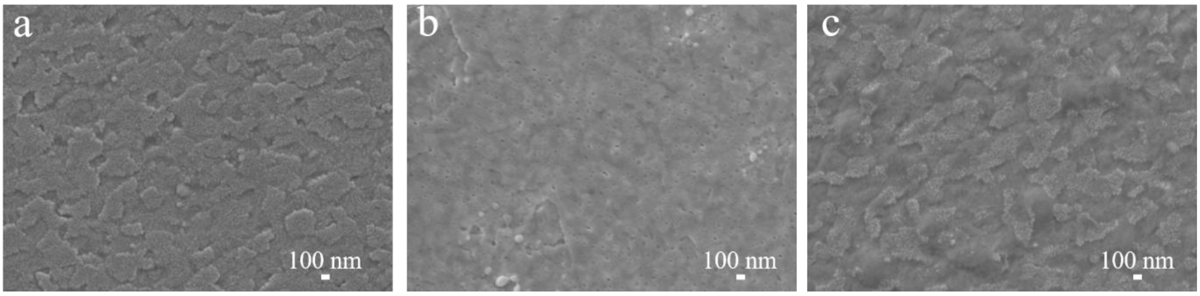

Figure S14. The SEM images of (**a**) ITO, (**b**) ITO/Cs_2CO_3 and (**c**) ITO/Cs_2CO_3 after deionized water washing.

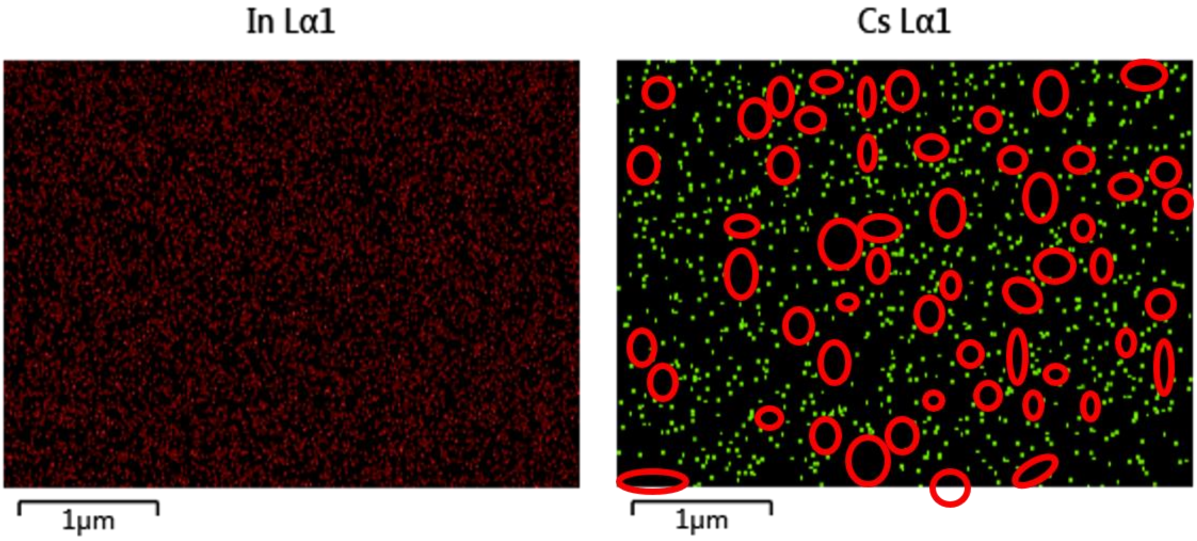

Figure S15. The EDS image of ITO/Cs_2CO_3 after deionized water washing. The red circle circles the area without Cs element.
